# Enhanced UV Photoresponsivity of ZnO Nanorods Decorated with Ag_2_S/ZnS Nanoparticles by Successive Ionic Layer Adsorption and Reaction Method

**DOI:** 10.3390/nano11020461

**Published:** 2021-02-11

**Authors:** Yimin Jin, Shujie Jiao, Dongbo Wang, Shiyong Gao, Jinzhong Wang

**Affiliations:** School of Materials Science and Engineering, Harbin Institute of Technology, Harbin 150001, China; jym921023@foxmail.com (Y.J.); gaoshiyong@hit.edu.cn (S.G.); jinzhong_wang@hit.edu.cn (J.W.)

**Keywords:** ZnO, Ag_2_S, successive ionic layer adsorption and reaction, photodetector

## Abstract

Recently, different kinds of energy band structures have been utilized to improve the photoelectric properties of zinc oxide (ZnO). In this work, ZnO nanorods were prepared by the hydrothermal method and then decorated with silver sulfide (Ag_2_S)/zinc sulfide (ZnS) via two-step successive ionic layer adsorption and reaction method. The photoelectric properties of nanocomposites are investigated. The results show that ZnO decorated with Ag_2_S/ZnS can improve the photocurrent of photodetectors from 0.34 to 0.56 A at bias of 9 V. With the immersion time increasing from 15 to 60 minutes, the photocurrent of photodetectors increases by 0.22 A. The holes in the valence band of ZnO can be transferred to the valence band of ZnS and Ag_2_S, which promotes the separation and suppresses the recombination of hole-electron pairs generated in ZnO. Moreover, electrons excited by ultraviolet (UV) light in Ag_2_S can also be injected into the conduction band of ZnO, which causes the photocurrent to increase more than the ZnO photodetector.

## 1. Introduction

Recently, increasing demand for clean energy, portable electronics, space and astronomical research, optical communications, and fire monitoring has caused UV photodetectors to be the subject of considerable attention due to their good flexibility, low-cost fabrication, and high sensitivity [1,2,3,4,5,6]. Many wide-bandgap semiconductors associated with this type of photodetectors have been explored [5,7,8,9,10,11,12]. Among wide-bandgap materials, zinc oxide has become a good candidate for UV photodetection due to its wide bandgap (3.37 eV), high exciton binding energy (60 meV), high chemical and thermal stability, low cost, and strong emission at room temperature [13,14,15,16]. To improve the properties of ZnO, incorporating noble metal nanostructures, compounding with carbon nanomaterials, and decorating with semiconductor quantum dots are effective approaches [4,17,18,19]. With decoration of semiconductors with different band structures, holes or electrons can be transferred from ZnO, which promotes separation and suppresses the recombination of hole-electron pairs generated in ZnO [20,21,22,23]. Ag_2_S as a direct, narrow bandgap (1.1 eV) semiconductor with optical absorption similar to silicon has attracted much attention as a promising candidate for photocatalysis and photoconduction [24,25,26]. Studies also proved that after accompanying Ag_2_S with ZnO, type-Ⅱ heterojunctions are formed. Due to the different valence bands of Ag_2_S and ZnO, the holes in the valence band of ZnO can be transferred to the valence band of Ag_2_S. This process can promote separation and suppress the recombination of hole-electron pairs generated in ZnO, leading to the improved optoelectrical properties of ZnO [27,28,29,30]. Recently, Li and colleagues prepared Ag_2_S-coupled ZnO microspheres with 1.68 μA/cm^2^ at 0.2 V under visible light [31]. Chen and colleagues prepared a Ag_2_S/ZnO core-shell nanoheterojunction with high photosensitivity in the wide spectral range from 400 to 1100 nm and a response time as short as 5 ms [32].

In this work, ZnO nanorods are prepared with the hydrothermal method. Then, ZnS and Ag_2_S are deposited via successive ionic layer adsorption and reaction method on the surface of ZnO nanorods. ZnS is an n-type [33,34,35] material, and Ag_2_S is a p-type [36,37,38,39] material. The nanocomposites demonstrate potential application in the fields of photodetection, photocatalysis, and solar cells [31,40,41,42]. In this work, the optoelectrical properties of nanocomposites are investigated.

## 2. Materials and Methods

The ZnO nanomaterials in this paper were prepared by the hydrothermal method on glass substrates with a conductive thin film of indium-doped tin oxides (ITO) on one side. The size of substrates was 1 × 1 cm. Before preparation, the substrates were cleaned with ultrasound successively in acetone, ethanol, and deionized water for 30 min. To prepare ZnO seed layers on ITO substrates by the sol-gel dip-coating method, the substrates were immersed in precursor solution for 15 min, and then, the samples were dried for 15 min. This process was repeated six times. Finally, an annealing treatment was performed in air at 150 °C for 30 min. Then ZnO nanorod arrays were formed on the substrates in a solution consisting of 0.03 M zinc acetate dehydrate (Zn(AC)_2_·2H_2_O) and 0.03 M hexamethylenetetramine (HMT) at 90 °C for 4 h. 

S^2−^ was incorporated into ZnO to form ZnS by immersing ZnO samples in aqueous solution containing 0.02 M Na_2_S for 5 min, 15 min, 30 min, 45 min, and 60 min, respectively and rinsing with pure ethanol. The ZnO nanorod arrays were decorated with Ag_2_S quantum dots (QDs) through the facile successive ionic layer adsorption and reaction (SILAR) method. ZnO samples were successively immersed in two different aqueous solutions, one containing 0.02 M Na_2_S and the other one containing 0.02 M AgNO_3_ aqueous solution for 30 min and different time, respectively. During immersion, the solution should be stirred. After immersion, the samples were rinsed with pure ethanol to remove excess precursors and blown dry at room temperature. Then, the Ag_2_S-modified samples were fabricated as metal-semiconductor-metal (MSM) photodetectors with indium (In) electrode. Electrodes were exploited to form ohmic contact between them and the nanocomposites. One electrode was prepared on the ZnO seed layer and the other one was prepared on the top of nanorod arrays. 

Surface morphologies of the nanocomposites were characterized using scanning electron microscopy (SEM, HITACH SU70, Tokyo, Japan). Finer details of the nanocomposites were characterized using transmission electron microscopy (TEM, FEI, Hillsboro, OR, USA) and high-resolution transmission electron microscopy (HRTEM, FEI, Hillsboro, OR, USA). The composition and bond band properties of the samples were measured by X-ray photoelectric spectroscopy (XPS, ESCALAB 250Xi, Thermo Fisher, Waltham, MA, USA). Ultraviolet–visible spectroscopy (Shimadzu UV1700-visible spectrophotometer) was utilized to characterize the optical properties. I–V characterization of the as-synthesized devices was measured by an electrochemical workstation (CHI660e, Chenhua instruments Ins., Shanghai, China) with a three-electrode system under UV led (λ = 365 nm). The photoresponsivity spectrum of the devices was obtained by measuring the photocurrent (calibrated with a standard Si photodiode) under the illumination of a UV-enhanced Xe lamp spectrum from 300 to 600 nm using a scanning monochromator (DSR600, Zolix, Beijing, China). 

## 3. Results and Discussion

**Figure 1:**
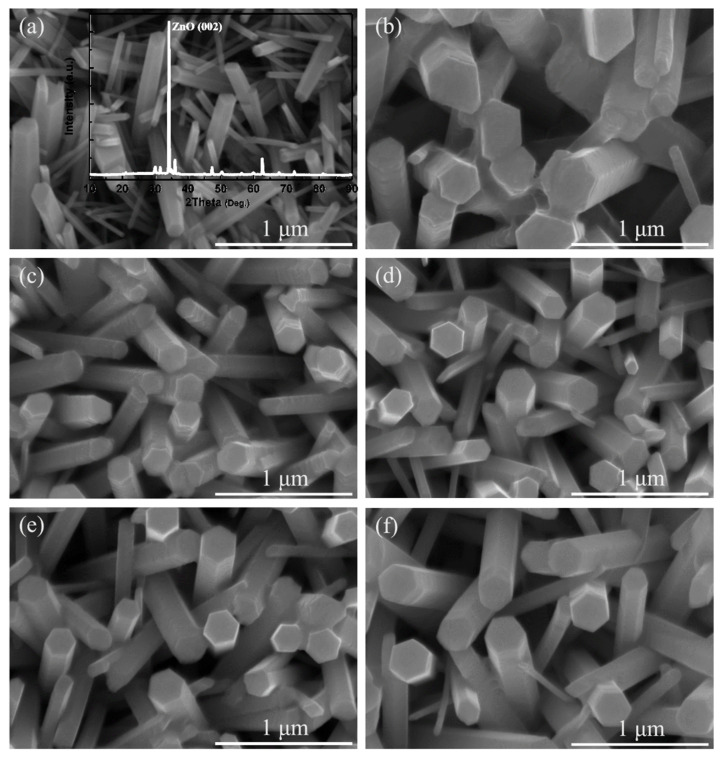
(**a**)–(**f**) SEM images of ZnO and ZnS/ZnO nanocomposites with different immersion time of 5, 15, 30, 45, and 60 min, respectively.

**Figure 2:**
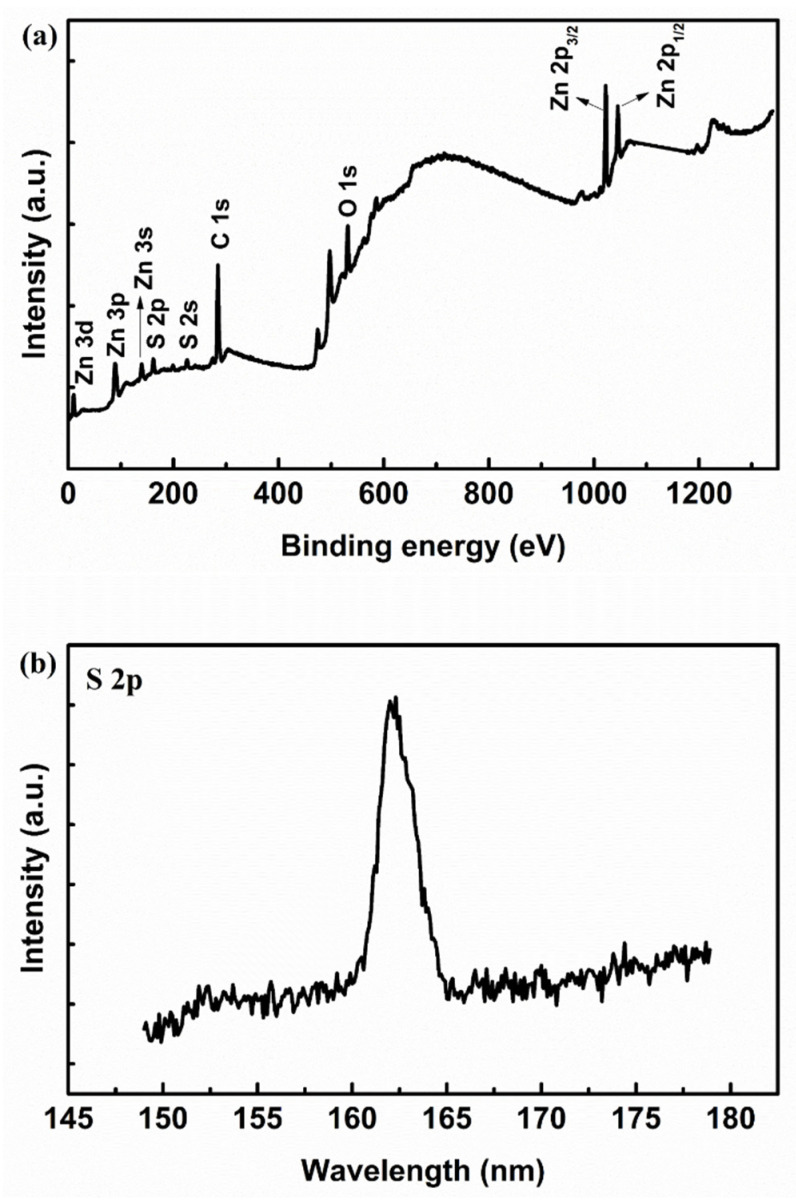
(**a**) XPS wide-survey spectrum; (**b**) S peak of ZnS/ZnO nanocomposites with the immersion time of 30 min.

The morphologies of ZnO and ZnS/ZnO heterojunctions array prepared by immersing ZnO samples in Na_2_S solution for 5, 15, 30, 45, and 60 min, respectively, are shown in Figure 1a–f. The hexagonal nanorods are not uniform, and the dominant diameter of the nanorods is about 260 nm. The XRD spectrum of the as-synthesized samples is shown in the inset of Figure 1a. Typical peaks belong to the wurtzite hexagonal phase of ZnO (JCPDS 36-1451), as presented in Figure 1a. When ZnO nanorods are immersed in Na_2_S solution, as shown in Figure 1b–f, the solution provides sulfide ions to react with zinc ions dissolved from the ZnO nanorods in order to form ZnS. During this process, the concentration of sulfide ions can be adjusted to influence the formation of ZnS. However, if the concentration is too large, many defects can be formed on the nanocomposites, leading to decreased efficiency. The formation of a ZnS shell can be determined by XPS spectra in Figure 2. The XPS wide-survey spectrum of the sample with an immersion time of 30 min is shown in Figure 2a. The characteristic peaks in the XPS spectrum can be assigned as Zn, S, C, or O, respectively. The ratio of S/Zn is about 0.16. No other impurity peaks can be found, showing that the obtained sample is of high purity. For ZnS/ZnO in Figure 2b, the S peak located at 162 eV corresponds to S 2p from ZnS. The above XPS analysis demonstrates the process of the formation of ZnS after immersion. Therefore, ZnO nanorods were covered by the ZnS shell through immersion.

**Figure 3:**
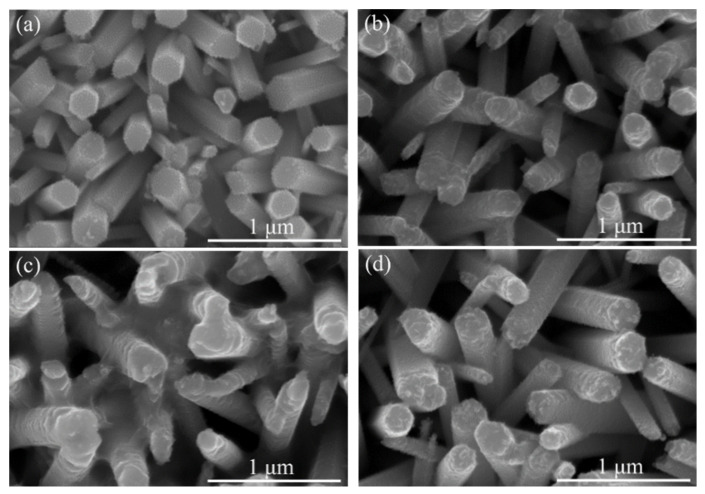
(**a**)–(**d**) SEM images of Ag_2_S/ZnO nanocomposites with different immersion time of 15, 30, 45, and 60 min, respectively.

Based on the ZnS/ZnO nanocomposites, the ZnS/ZnO samples with the immersion time of 30 min in Na_2_S solution were then immersed in 0.02 M AgNO_3_ aqueous solution for 15, 30, 45, and 60 min, respectively, which allows for the Ag_2_S/ZnS/ZnO nanocomposites to be obtained. The morphologies of the as-synthetized Ag_2_S/ZnS/ZnO nanorod arrays are shown in Figure 3a–d. It can be seen that after immersion, the nanorods were covered with spherical nanoparticles at the short immersion time. Because of the difference between the solubility product constant (K_sp_) of ZnS (2.93 × 10^−25^) and K_sp_ of Ag_2_S (6.69 × 10^−50^) [43], the cation exchange process occurs where zinc ions are replaced by silver ions, leading to the formation of Ag_2_S, which indicates that the spherical nanoparticles are Ag_2_S. With the increasing immersion time, the nanorods demonstrate a complete shape change from hexagon to ellipse. These results suggest that Ag_2_S was successfully deposited onto the surface of the nanorods.

**Figure 4:**
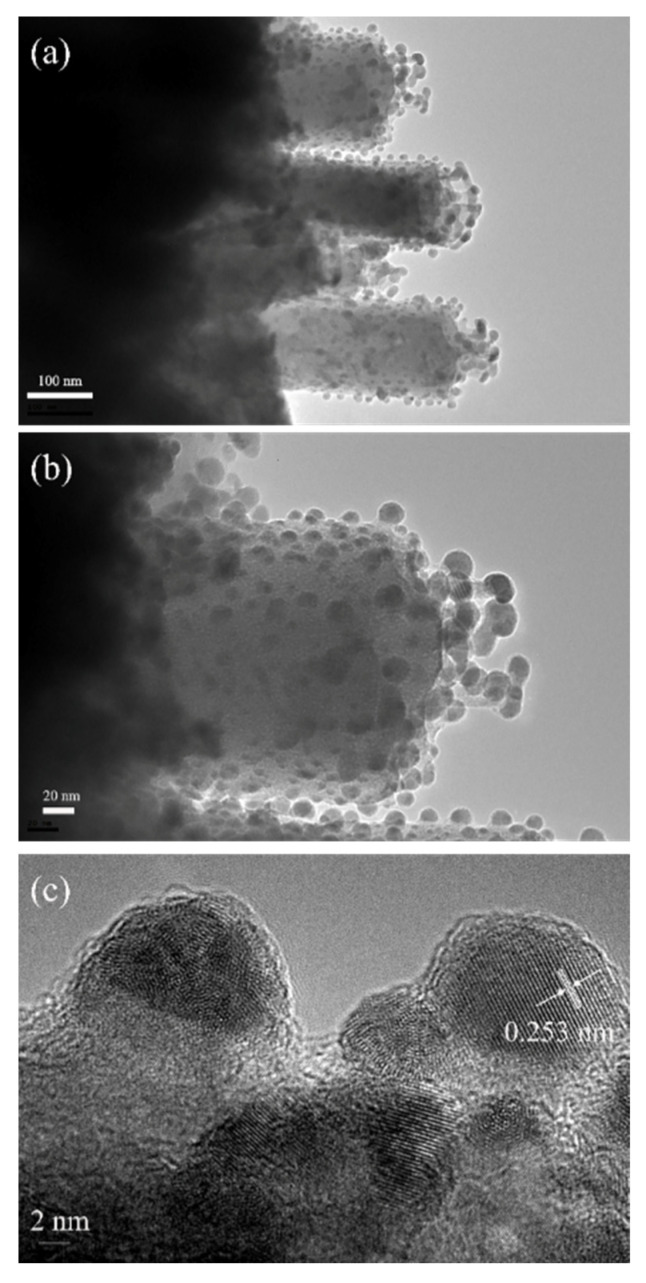
(**a**) and (**b**) TEM images of Ag_2_S/ZnS/ZnO nanocomposites with an immersion time of 60 min; (**c**) HRTEM image of nanoparticles.

In order to confirm the formation of Ag_2_S/ZnS/ZnO composites, TEM and HRTEM measurements were performed on the nanocomposite with the immersion time of 60 min, as illustrated in Figure 4. A large number of quantum dots evenly deposited onto the nanorod surface is shown in Figure 4a,b, which show that densely distributed QDs are formed on the surface of nanorods, and the diameter of the quantum dots is about 10–15 nm. Figure 4c shows the HRTEM image of the quantum dots deposited on the nanorod surface. It can be observed that the quantum dots have a spherical shape with a diameter of about 10 nm. The obvious lattice arrangement can be found in the quantum dots, and the d-spacing estimated to be 0.253 nm is indexed to the (−103) orientation of Ag_2_S crystalline.

**Figure 5:**
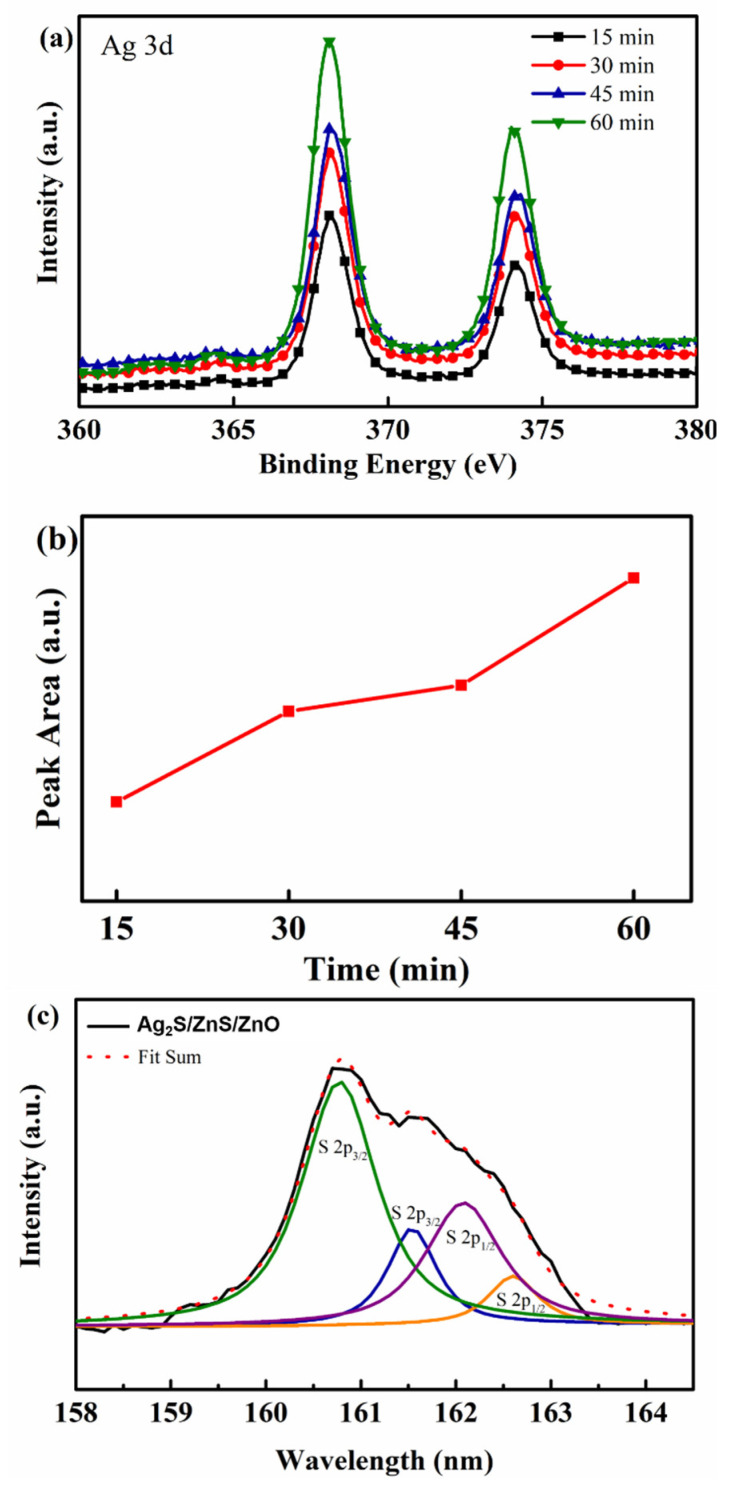
(**a**) XPS spectra of Ag 3d for Ag_2_S/ZnS/ZnO nanocomposites with various immersion time; (**b**) peak area of Ag 3d_5/2_ with different immersion time; (**c**) XPS spectra of S 2p for Ag_2_S/ZnS/ZnO nanocomposites with the immersion time of 60 min.

To further investigate the structures of Ag_2_S/ZnS/ZnO nanocomposites, XPS measurements were measured. Figure 5a shows the Ag 3d region of the XPS spectra. The peak position of Ag 3d_5/2_ is located at about 368 eV. This value is in good agreement with the reported values for Ag_2_S. Then, the peak area of Ag elements with different immersion time was calculated, as shown in Figure 5b. It is obvious that the Ag concentration increases with the increasing immersion time. Meanwhile, according to the XPS spectrum, the Ag/Zn ratio increasing from 0.08 to 0.22 indicates the same results. This result is consistent with the SEM images shown in Figure 3. Figure 5c shows the S 2p region of the XPS spectrum with the immersion time of 60 min. The black line represents the experimental data, and the red dots correspond to the fitted curve. Four labelled fitting Gaussian peaks were used to fit the experimental data. The binding energy of the S 2p_3/2_ peak located at 161.5 eV is in accordance with the binding energy of ZnS. The lowest energy peak of S 2p_3/2_ is located at 160.93 eV, which corresponds to Ag_2_S [44,45,46,47]. These results illustrate that Ag_2_S/ZnS/ZnO nanocomposites were formed after immersion.

**Figure 6:**
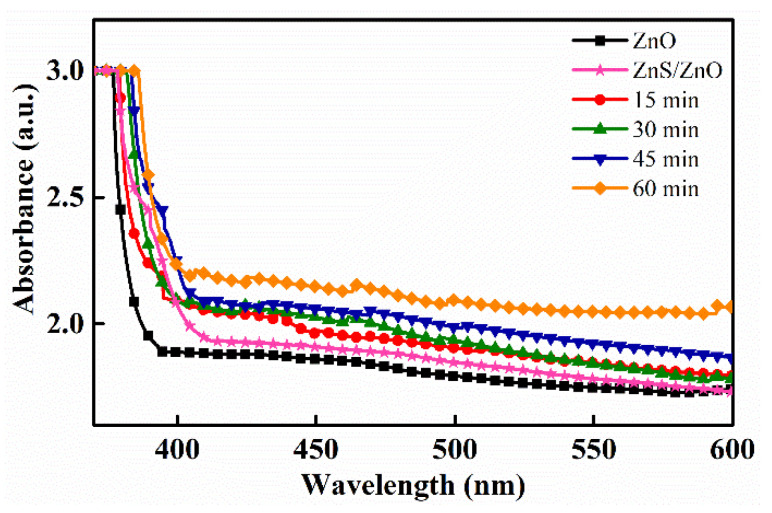
Absorption spectra of Ag_2_S/ZnS/ZnO nanocomposites with various immersion time.

To investigate the optical properties of Ag_2_S/ZnS/ZnO nanocomposites, UV-vis absorption spectra from 350 to 600 nm were examined. For comparison, the spectrum of pure ZnO nanorods was also measured, indicated by the black line. Figure 6 shows the UV-vis absorption spectra of Ag_2_S/ZnS/ZnO nanocomposites with various immersion time, revealing that the absorption edge of ZnO is extended to the visible region by decoration with Ag_2_S because of its narrow bandgap of ~1.1 eV. When visible light is observable on the nanocomposites, hole-electron pairs are generated in Ag_2_S, leading to the absorption in visible light. With the increasing immersion time, more amounts of Ag_2_S are deposited, thereby causing the increase in absorption in the visible light region.

**Figure 7:**
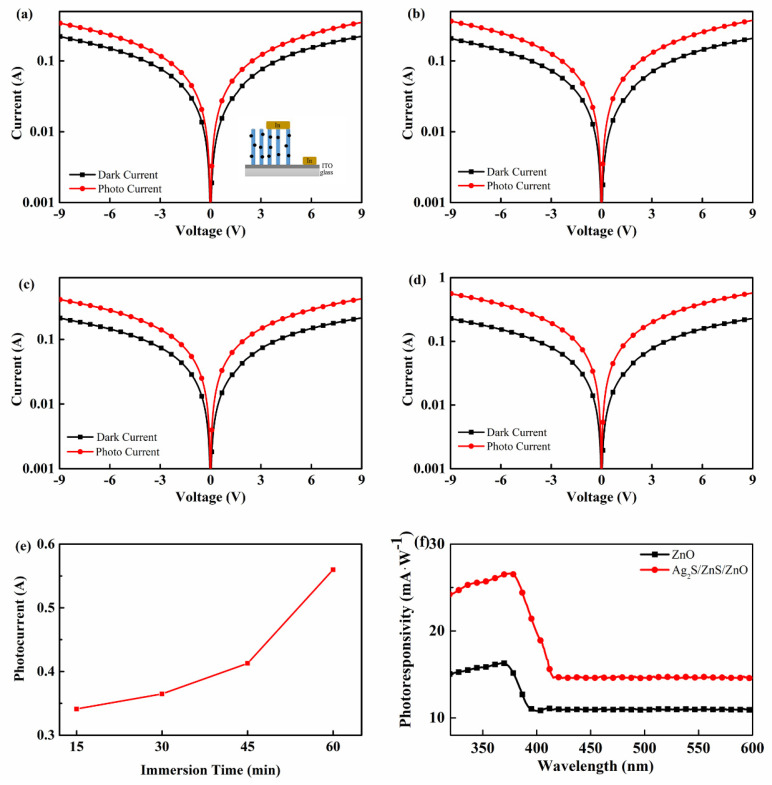
(**a**)–(**d**) I-V characteristics of photodetectors with various immersion time ((**a**) 15 min, (**b**) 30 min, (**c**) 45 min, and (**d**) 60 min) under UV light; the figure inset (**a**) is a schematic diagram of the photodetector; (**e**) photocurrent of photodetectors with various immersion time under 9 V bias; (**f**) photoresponsivity of the photodetector with the immersion time of 60 min.

A photodetector was fabricated with In electrodes to investigate the optoelectrical properties of Ag_2_S/ZnS/ZnO nanocomposites. The I-V characteristics of photodetectors with various immersion time under 365 nm UV LED are shown in Figure 7a–d. It can be observed that photodetectors have a photoresponse under UV illumination. The reverse current is high and of the same order of magnitude as the direct current, demonstrating that photoconductive photodetectors were fabricated. At the same voltage, the photocurrent of photodetectors with various immersion time changes from 0.34 to 0.56 A under UV illumination. Meanwhile, the dark current of the photodetectors, which can be ascribed to oxygen vacancy in ZnO, is 0.22, 0.20, 0.21, and 0.22 A, respectively. Thus, the on–off ratio of the photodetectors at a bias of 9 V is 1.53, 1.77, 1.94, and 2.47, respectively. Figure 7e shows the photocurrent of photodetectors with various immersion time under 9 V bias. It is observed that the photocurrent increases with the increase in immersion time. This phenomenon can be attributed to the replacement of ZnS by Ag_2_S. The photoresponsivity of the photodetector with the immersion of 60 min is shown in Figure 7f. It can be observed that the photoresponsivity increases for the Ag_2_S/ZnS/ZnO nanocomposites when compared with pure ZnO photodetectors not only in UV regions but also in the visible wavelength region. In addition, the UV-to-visible rejection ratio was improved from 1.48 to 1.82.

**Figure 8:**
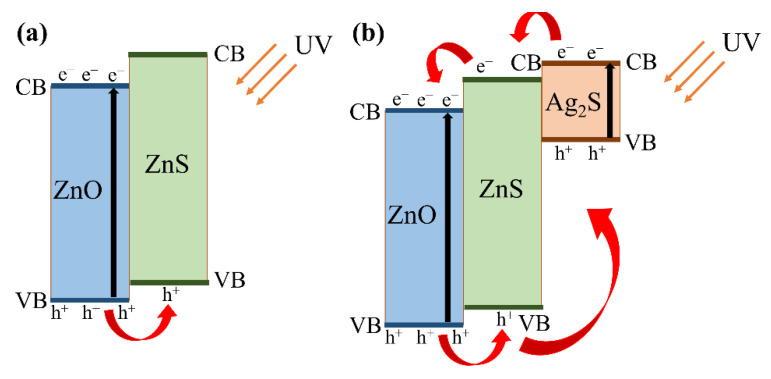
Energy band schematic diagrams of different nanocomposites: (**a**) ZnS/ZnO; (**b**) Ag_2_S/ZnS/ZnO.

In order to explain the mechanism of photoresponse of Ag_2_S/ZnS/ZnO nanocomposites, the energy band schematic diagrams of different nanocomposites are shown in Figure 8. Considering the fact that not all amounts of ZnS are replaced by Ag_2_S, there are two types of energy bands in nanocomposites [48,49]. Figure 8a shows the energy band schematic diagrams of ZnS/ZnO. Under the illumination of 365 nm UV light, electrons are excited from the valence band to the conduction band of ZnO, resulting in an increase in the photocurrent. Because of the different valence bands of ZnS and ZnO, the holes in the valence band of ZnO can be transferred to the valence band of ZnS. This process can promote the separation of photogenerated hole-electron pairs and suppress their recombination in ZnO. Meanwhile, because the photon energy of UV light is smaller than the bandgap of ZnS, no electrons are excited to the conduction band in ZnS. Figure 8b shows the energy band schematic diagrams of Ag_2_S/ZnS/ZnO nanocomposites. It can be seen that the cascade structure represents the stepwise positions of band edges via the redistribution of ZnS and Ag_2_S in order to align Fermi levels. This structure is suitable for the injection of photogenerated electrons from Ag_2_S to ZnO and the transfer of holes from ZnO to Ag_2_S, and it is advantageous for the separation and transmission of hole-electron pairs. Generally, this structure can further increase the photocurrent compared with the ZnS/ZnO structure because of the injection of electrons. With the increasing immersion time, the first type is gradually replaced by the second type, which further increases the photocurrent. These results are consistent with the I–V characteristics of Ag_2_S/ZnO/ZnO nanocomposites with different immersion time.

## 4. Conclusions

In summary, Ag_2_S/ZnS/ZnO nanocomposites were prepared on ITO substrates via two-step facile successive ionic layer adsorption and reaction method with different immersion time. SEM and TEM images illustrate that the ZnS and Ag_2_S were evenly deposited on ZnO nanorods. The optical properties of Ag_2_S/ZnS/ZnO nanocomposites were investigated by UV-vis absorption spectra, which show that the absorption of Ag_2_S/ZnS/ZnO nanocomposites was extended to the visible light region due to the narrow bandgap of Ag_2_S. Then MSM photodetectors were fabricated. The influence of ZnS and Ag_2_S on the photocurrent of the photodetectors was investigated. The photocurrent increased with the increasing immersion time of AgNO3 solutions due to the increasing electrons injected from Ag_2_S into ZnO. The energy band schematic diagrams were used to explain the photoresponse of the photodetectors. The transfer of holes and the injection of electrons can both enhance the photoresponse compared with pure ZnO. Compared to other similar systems, the nanocomposites improved the photocurrent under UV illumination and demonstrate potential applications in other fields [50,51,52].

## Data Availability

The data presented in this study are available on request from the corresponding author.
